# Resistance Exercise Improves Glycolipid Metabolism and Mitochondrial Biogenesis in Skeletal Muscle of T2DM Mice via miR-30d-5p/SIRT1/PGC-1α Axis

**DOI:** 10.3390/ijms252212416

**Published:** 2024-11-19

**Authors:** Lifang Zheng, Zhijian Rao, Jiabin Wu, Xiaojie Ma, Ziming Jiang, Weihua Xiao

**Affiliations:** 1 College of Physical Education, Shanghai University, Shanghai 200444, China; zhenglf21@shu.edu.cn (L.Z.); 18621799685@163.com (X.M.); shujzm@shu.edu.cn (Z.J.); 2 College of Physical Education, Shanghai Normal University, Shanghai 200234, China; raoz19@shnu.edu.cn; 3 Shanghai Key Laboratory of Human Performance, Shanghai University of Sport, Shanghai 200438, China; wujiabin1125@163.com; 4 The Key Laboratory of Exercise and Health Sciences of Ministry of Education, Shanghai University of Sport, Shanghai 200438, China

**Keywords:** resistance exercise, miRNAs, skeletal muscle, insulin resistance, T2DM

## Abstract

Exercise is a recognized non-pharmacological treatment for improving glucose homeostasis in type 2 diabetes (T2DM), with resistance exercise (RE) showing promising results. However, the mechanism of RE improving T2DM has not been clarified. This study aims to investigate the effects of RE on glucose and lipid metabolism, insulin signaling, and mitochondrial function in T2DM mice, with a focus on the regulatory role of miR-30d-5p. Our results confirmed that RE significantly improved fasting blood glucose, IPGTT, and ITT in T2DM mice. Enhanced expression of IRS-1, p-PI3K, and p-Akt indicated improved insulin signaling. RE improved glycolipid metabolism, as well as mitochondrial biogenesis and dynamics in skeletal muscle of T2DM mice. We also found that miR-30d-5p was upregulated in T2DM, and was downregulated after RE. Additionally, in vitro, over-expression of miR-30d-5p significantly increased lipid deposition, and reduced glucose uptake and mitochondrial biogenesis. These observations were reversed after transfection with the miR-30d-5p inhibitor. Mechanistically, miR-30d-5p regulates glycolipid metabolism in skeletal muscle by directly targeting SIRT1, which affects the expression of PGC-1α, thereby influencing mitochondrial function and glycolipid metabolism. Taken together, RE effectively improves glucose and lipid metabolism and mitochondrial function in T2DM mice, partly through regulating the miR-30d-5p/SIRT1/PGC-1α axis. miR-30d-5p could serve as a potential therapeutic target for T2DM management.

## 1. Introduction

Diabetes mellitus (DM) affects over 450 million people worldwide, with 90–95% suffering from type 2 diabetes mellitus (T2DM) [1]. This chronic condition is associated with various complications such as diabetic cardiomyopathy, nephropathy, and retinopathy, posing significant economic burdens on individuals and society [2]. A hallmark of T2DM is insulin resistance (IR), characterized by decreased sensitivity of insulin target organs, notably skeletal muscles, crucial for glucose uptake and metabolism.

Current guidelines recommend regular aerobic exercise and resistance exercise (RE) to manage T2DM [3]. Aerobic exercise’s benefits are well-documented. The previous study indicated that RE improved whole body and skeletal muscle insulin sensitivity in the offspring of overweight/obese mothers [4]. In addition, an 8-week RE regimen accelerated GLUT4 translocation, protein kinase B (Akt) and protein kinase C (PKC) phosphorylation, and elevation of muscular DHT (5α-dihydrotestosterone) levels, improving fasting blood glucose and insulin sensitivity in T2DM rats [5,6]. However, the mechanism by which RE improves blood glucose levels and insulin sensitivity in T2DM patients has not been fully elucidated.

Recent research has highlighted microRNAs (miRNAs) as key regulators in modulating insulin resistance and metabolic pathways in skeletal muscle [7,8,9]. miR-30d-5p, for instance, has been implicated in regulating glucose metabolism by targeting the GLUT4 signaling pathway and insulin gene expression [10]. Moreover, SIRT1, a validated target of miR-30d-5p [11], is known to modulate PGC-1α [12,13], a critical regulator of mitochondrial biogenesis and fatty acid β-oxidation. The interplay between miR-30d-5p, SIRT1, and PGC-1α in skeletal muscle under conditions of insulin resistance remains an area of active investigation.

Given the significant impact of insulin resistance on glycolipid metabolism and mitochondrial function in skeletal muscle, understanding the role of miR-30d-5p in these processes could provide valuable insights into T2DM management strategies. However, the specific effects of RE on miR-30d-5p and its downstream targets in the skeletal muscle of T2DM mice remain underexplored. This study aims to investigate whether RE improves glycolipid metabolism and mitochondrial function in T2DM mouse skeletal muscle through the miR-30d-5p/SIRT1/PGC-1α axis.

## 2. Results

### 2.1. Resistance Exercise Improved Body Composition and Glucose Handling in T2DM Mice

**Figure 1:**
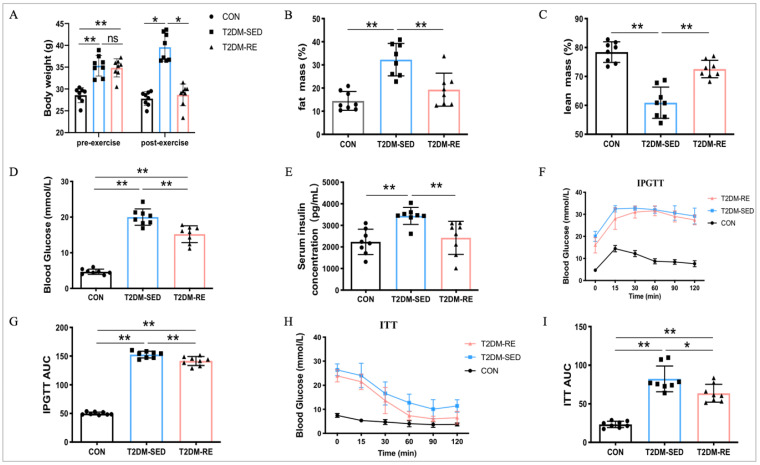
Resistance exercise improved body composition and glucose handling in T2DM mice. (**A**) Body weight; (**B**) fat mass (%); (**C**) lean mass (%); (**D**) fasting blood glucose; (**E**) serum insulin concentration; (**F**) plot for glucose tolerance tests (IPGTT, 1g/kg BW) in overnight fasted mice; (**G**) AUC values confirmed impairment of glucose tolerance in T2DM mice; (**H**) plots for insulin tolerance tests (ITT, 1IU/kg BW) in mice fasted for 6h; (**I**) AUC values confirmed impairment of insulin tolerance in T2DM mice. All data are presented as mean ± SD. *n* = 8 per group; * *p* < 0.05, ** *p* < 0.01.

The mice were fed a high-fat diet for 12 weeks and injected intraperitoneally with STZ to induce T2DM. Subsequently, T2DM mice underwent resistance exercise for 8 weeks. Before exercise, compared to the control (CON) mice, the body weight of mice in both the T2DM-SED and T2DM-RE group was significantly increased, with no significant difference between the T2DM-SED and T2DM-RE groups (Figure 1A). After the exercise intervention, the body weight of T2DM-SED mice remained significantly higher than that of CON mice and T2DM-RE mice (Figure 1A). Additionally, RE increased lean mass and decreased fat mass in T2DM mice (Figure 1B,C). These results suggest that an 8-week RE regimen improves body weight and body composition in T2DM mice.

Furthermore, T2DM-SED mice exhibited higher fasting blood glucose and serum insulin concentrations compared to CON mice. However, RE significantly reduced fasting blood glucose and serum insulin concentrations in T2DM mice after the 8-week RE intervention (Figure 1D,E). We also assessed the impact of RE on glucose homeostasis and found that T2DM mice displayed higher glucose intolerance and insulin intolerance compared to CON mice (Figure 1F–I). In contrast, RE treatment mitigated T2DM-induced impairments in glucose homeostasis, improving both glucose tolerance and insulin tolerance compared to T2DM-SED mice (Figure 1F–I).

### 2.2. RE Improved Insulin Sensitivity and Glucose Metabolism in Skeletal Muscle of T2DM Mice

**Figure 2:**
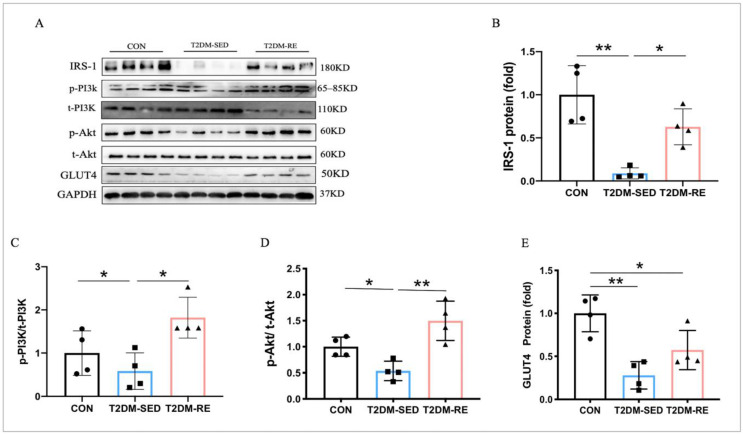
RE improved insulin sensitivity and glucose metabolism in the skeletal muscle of T2DM mice. (**A**) Western blot results revealed protein levels of IRS-1, p-PI3K/t-PI3K, p-Akt/t-Akt, and GLUT4 in skeletal muscle; (**B**–**E**) quantification of IRS-1, p-PI3K/t-PI3K, p-Akt/t-Akt, and GLUT4 expression levels presented in (**A**). All data are presented as mean ± SD. *n* = 3 per group; * *p* < 0.05, ** *p* < 0.01.

Skeletal muscle is the primary site for glucose uptake and utilization. IRS-1 is central to blood glucose regulation, activating the downstream PI3K/Akt signaling pathway. Compared with CON mice, the protein expression of IRS-1, p-PI3K, and p-Akt was significantly lower in the skeletal muscle of T2DM mice but was upregulated by RE (Figure 2A–D). These results indicate that type 2 diabetes inhibits the insulin signaling pathway in skeletal muscle, leading to insulin resistance. In contrast, RE activates the insulin signaling pathway and improves insulin sensitivity in the skeletal muscle of T2DM mice. Additionally, GLUT4 translocation to the cell membrane facilitates glucose uptake. As shown in Figure 2A,E, the reduced level of GLUT4 protein impairs glucose uptake in skeletal muscle. However, RE reverses the suppression of GLUT4 protein expression in the skeletal muscle of T2DM mice. Thus, resistance exercise significantly enhances skeletal muscle insulin sensitivity and glucose metabolism by activating the insulin signaling pathway and GLUT4 in T2DM mice.

### 2.3. RE Promoted Lipid Metabolism in Skeletal Muscle of T2DM Mice

**Figure 3:**
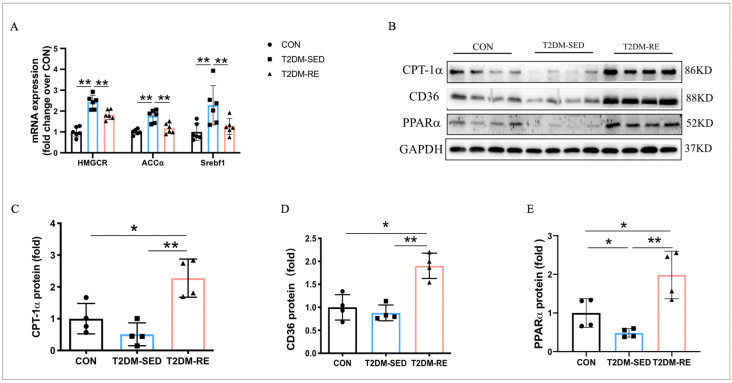
RE promoted lipid oxidation and transport and inhibited lipid synthesis in skeletal muscle of T2DM mice. (**A**) Relative mRNA expression of HMGCR, ACCα, and Srebf1 measured by qRT-PCR; (**B**) Western blot results revealed protein levels of CPT-1α, CD36, PPARα in skeletal muscle; (**C**–**E**) quantification of CPT-1α, CD36, and PPARα expression levels presented in (**A**). All data are presented as mean ± SD. *n* = 4 per group; * *p* < 0.05, ** *p* < 0.01.

ACCα, HMGCR, and Srebf1 are key genes related to lipid synthesis. As shown in Figure 3A, diabetes significantly increases the mRNA expression of ACCα, HMGCR, and Srebf1, while RE significantly reduces the mRNA expression of these genes in the skeletal muscle of T2DM-RE mice compared to T2DM-SED mice. These results suggest that RE inhibits lipogenesis in the skeletal muscle of T2DM mice. CPT-1α, PPARα, and CD36 are involved in the regulation of lipid oxidation and transport. Compared to CON mice, the protein expression of PPARα was significantly decreased in T2DM mice, while RE significantly increased the protein expression of CPT-1α, PPARα, and CD36 (Figure 3B–E), indicating that RE enhances lipid oxidation and transport in the skeletal muscle of T2DM mice.

### 2.4. RE Enhanced Mitochondrial Biogenesis and Dynamics in the Skeletal Muscle of T2DM Mice

**Figure 4:**
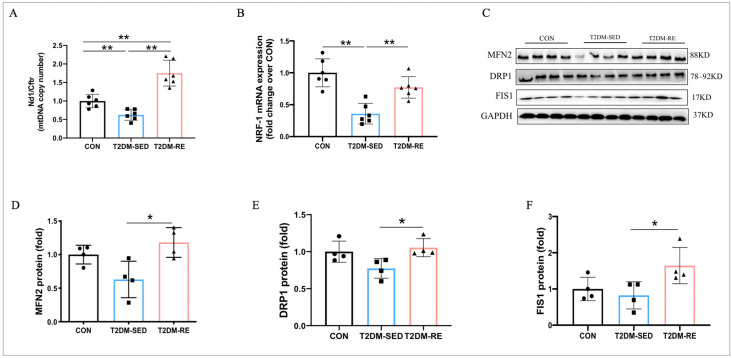
RE enhanced mitochondrial biogenesis and dynamics in the skeletal muscle of T2DM mice. (**A**) Mitochondrial DNA copy number; (**B**) relative mRNA expression of NRF-1 measured by qRT-PCR; (**C**) Western blot results revealed protein levels of MFN2, DRP1, and FIS1 in skeletal muscle; (**D**–**F**) quantification of MFN2, DRP1, and FIS1 expression levels presented in (**C**). All data are presented as mean ± SD. *n* = 4 per group; * *p* < 0.05, ** *p* < 0.01.

Our results showed that the expression of NRF1 and mtDNA copy number were lower in T2DM mice compared to CON mice (Figure 4A,B). An 8-week RE regimen significantly enhanced the expression of NRF1 and mtDNA copy number in the skeletal muscle of T2DM mice (Figure 4A,B), suggesting that RE increases mitochondrial biogenesis through upregulating the NRF1/mtDNA signaling pathway. Mitochondrial fusion and fission are crucial for maintaining mitochondrial function. Therefore, we assessed the protein expression of MFN2, DRP1, and FIS1, critical components of mitochondrial dynamics. As shown in Figure 4C, the expression of DRP1, MFN2, and FIS1 did not significantly change compared to CON mice. However, RE treatment markedly enhanced the expression of DRP1, MFN2, and FIS1 in the skeletal muscle of T2DM-RE mice compared to T2DM-SED mice (Figure 4C–F).

### 2.5. RE Regulates the miR-30d-5p/SIRT1/PGC-1α Axis

**Figure 5:**
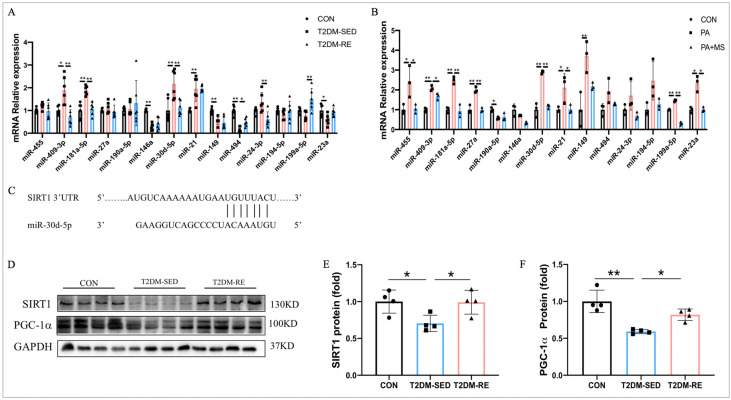
RE regulates the miR-30d-5p/SIRT1/PGC-1α axis. (**A**,**B**) Relative mRNA expressions of miR-455, miR-409-3p, miR-181a-5p, miR-27a, miR-190a-5p, miR-146a, miR-30d-5p, miR-21, miR-149, miR-494, miR-24-3p, miR-194-5p, miR-199a-5p, and miR-23a in C2C12 myotube cells/skeletal muscle; (**C**) TargetScan predicts target genes for miR-30d-5p; (**D**) Western blot results revealed protein levels of SIRT1 and PGC-1α in skeletal muscle; (**E**,**F**) quantification of SIRT1 and PGC-1α expression levels presented in (**D**). All data are presented as mean ± SD. *n* = 4 per group; * *p* < 0.05, ** *p* < 0.01.

Emerging evidence indicates that numerous miRNAs play important roles in insulin resistance and diabetes. We reviewed the literature related to skeletal muscle insulin resistance and then selected miRNAs that had unexplored mechanistic roles for validation in our model. Therefore, we examined the expression of 14 miRNAs in the skeletal muscle of T2DM mice and insulin-resistant C2C12 myotube cells using qRT-PCR. Additionally, we evaluated the effects of mechanical stretch (MS) and resistance exercise on the expression of these miRNAs. The results showed that miR-409-3p, miR-181a-5p, and miR-30d-5p were upregulated in insulin-resistant C2C12 myotube cells and the skeletal muscle of T2DM mice, while mechanical stretch and resistance exercise reversed these changes (Figure 5A,B). Furthermore, the changes in miR-30d-5p were the most significant in insulin-resistant C2C12 myotube cells and skeletal muscle (Figure 5A,B), suggesting that miR-30d-5p may play a vital role in improving glucose homeostasis in T2DM mice through resistance exercise.

We identified the potential target genes of miR-30d-5p using the TargetScan online database, revealing SIRT1 as a target gene (Figure 5C). Previous studies have demonstrated that miR-30d-5p binds to the 3′ UTR of SIRT1 [11]. SIRT1 is known to regulate PGC-1α. Our results indicated that the expression of SIRT1 and PGC-1α was significantly reduced in the skeletal muscle of T2DM mice compared to CON mice, while the expression of these proteins was elevated after 8 weeks of RE (Figure 5D–F). Collectively, these data indicate that RE improves glucose homeostasis in T2DM mice by regulating the miR-30d-5p/SIRT1/PGC-1α axis.

### 2.6. MiR-30d-5p Regulates Glucose Metabolism in C2C12 Myotube Cells

**Figure 6:**
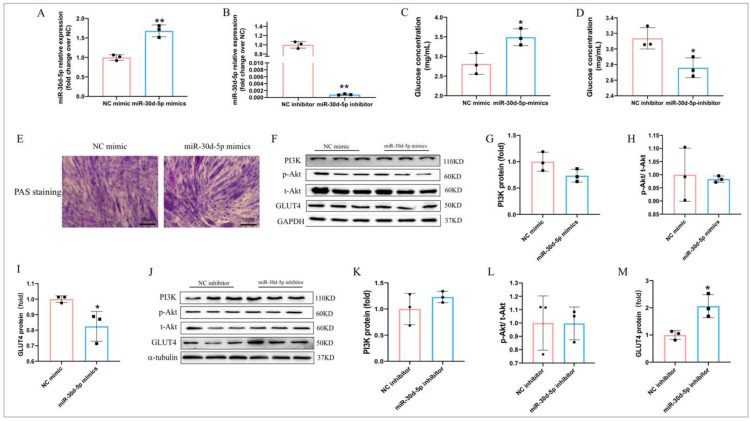
miR-30d-5p regulates glucose metabolism in C2C12 myotube cells. (**A**,**B**) miR-30d-5p relative expression after transfection with miR-30d-5p mimics/inhibitor; (**C**,**D**) glucose concentration after transfection with miR-30d-5p mimics/inhibitor; (**E**) PAS staining in C2C12 myotube cells after transfection with miR-30d-5p mimics; (**F**) Western blot results revealed protein levels of PI3K, p-Akt, t-Akt, and GLUT4 after transfection with miR-30d-5p mimics; (**G**–**I**) quantification of PI3K, p-Akt, t-Akt, and GLUT4 expression levels presented in (**F**); (**J**) Western blot results revealed protein levels of PI3K, p-Akt, t-Akt, and GLUT4 after transfection with miR-30d-5p inhibitor; (**K–M**) quantification of PI3K, p-Akt, t-Akt, and GLUT4 expression levels presented in (**J**). All data are presented as mean ± SD. *n* = 3 per group; Scale bars, 50 μm, * *p* < 0.05, ** *p* < 0.01.

We further explored whether miR-30d-5p regulates glucose metabolism in skeletal muscle. We transfected C2C12 myotubes with miR-30d-5p mimics and inhibitors. We found that 50 nM miR-30d-5p mimics greatly enhanced miR-30d-5p expression, whereas 100 nM miR-30d-5p inhibitor significantly decreased its expression compared to the negative control (Figure 6A,B). Additionally, miR-30d-5p mimics significantly inhibited glucose uptake in C2C12 myotubes compared to the control treatment (Figure 6C). In contrast, miR-30d-5p inhibitor significantly increased glucose uptake in C2C12 myotubes (Figure 6D). However, miR-30d-5p mimics did not affect glycogen synthesis in C2C12 myotube cells (Figure 6E). Our results indicate that miR-30d-5p mimics do not affect the PI3K/AKT pathway (Figure 6F–H), yet significantly inhibit GLUT4 expression (Figure 6I). Similarly, the miR-30d-5p inhibitor does not alter the PI3K/AKT pathway (Figure 6J–L), but it markedly increases GLUT4 expression (Figure 6M). These results indicate that miR-30d-5p regulates glucose metabolism in C2C12 myotube cells, suggesting its potential role in skeletal muscle insulin resistance.

### 2.7. MiR-30d-5p Regulates Lipid Metabolism in C2C12 Myotube Cells

**Figure 7:**
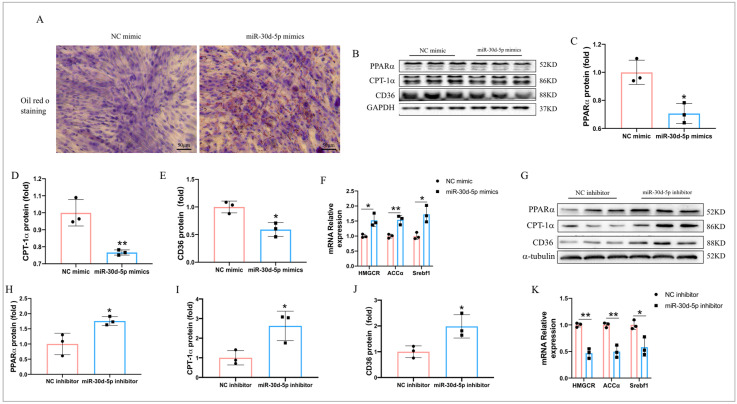
miR-30d-5p regulates lipid metabolism in C2C12 myotube cells. (**A**) Oil Red O staining in C2C12 myotube cells after transfection with miR-30d-5p mimics; (**B**) Western blot results revealed protein levels of PPARα, CPT-1α, and CD36 after transfection with miR-30d-5p mimics; (**C**–**E**) quantification of PPARα, CPT-1α, and CD36 expression levels presented in (**B**); (**F**) relative mRNA expression of HMGCR, ACCα, and Srebf1 after transfection with miR-30d-5p mimics; (**G**) Western blot results revealed protein levels of PPARα, CPT-1α, and CD36 after transfection with miR-30d-5p inhibitor. (**H**–**J**) quantification of PPARα, CPT-1α, and CD36 expression levels presented in (**G**); (**K**) relative mRNA expression of HMGCR, ACCα, and Srebf1 after transfection with miR-30d-5p inhibitor. All data are presented as mean ± SD. *n* = 3 per group; Scale bars, 50 μm, * *p* < 0.05, ** *p* < 0.01.

Compared to the control treatment, miR-30d-5p mimics significantly increased lipid deposition in C2C12 myotubes (Figure 7A). Moreover, transfection with miR-30d-5p mimics markedly suppressed the protein levels of PPARα, CPT-1α, and CD36, while promoting the mRNA levels of HMGCR, ACCα, and Srebf1 in C2C12 myotube cells (Figure 7B–F). In contrast, transfection with miR-30d-5p inhibitors dramatically enhanced the protein expression of PPARα, CPT-1α, and CD36, and decreased the mRNA levels of HMGCR, ACCα, and Srebf1 in C2C12 myotube cells (Figure 7G–K). These results indicate that miR-30d-5p affects lipid metabolism in C2C12 myotube cells by regulating the expression of PPARα, CPT-1α, CD36, HMGCR, ACCα, and Srebf1.

### 2.8. MiR-30d-5p Regulates Mitochondrial Biogenesis and Dynamics in C2C12 Myotube Cells

**Figure 8:**
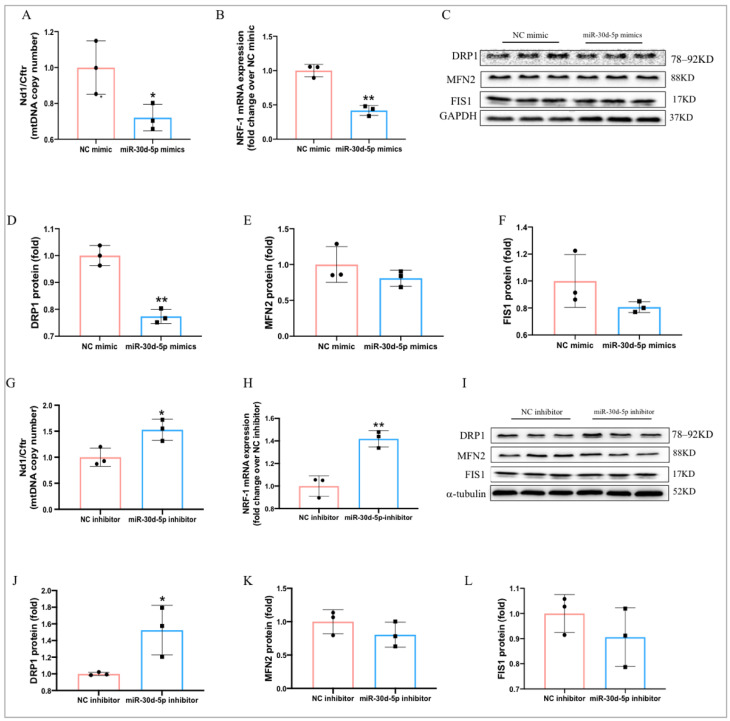
miR-30d-5p regulates mitochondrial biogenesis and dynamics in C2C12 myotube cells. (**A**) mtDNA copy number after transfection with miR-30d-5p mimics; (**B**) relative mRNA expression of NRF-1 after transfection with miR-30d-5p mimics; (**C**) Western blot results revealed protein levels of DRP1, MFN2, and FIS1 after transfection with miR-30d-5p mimics; (**D**–**F**) quantification of DRP1, MFN2, and FIS1 expression levels presented in (**C**); (**G**) mtDNA copy number after transfection with miR-30d-5p inhibitor; (**H**) relative mRNA expression of NRF-1 after transfection with miR-30d-5p inhibitor; (**I**) Western blot results revealed protein levels of DRP1, MFN2, and FIS1 after transfection with miR-30d-5p inhibitor; (**J**–**L**) quantification of DRP1, MFN2, and FIS1 expression levels presented in (**I**). All data are presented as mean ± SD. *n* = 3 per group; * *p* < 0.05, ** *p* < 0.01.

We examined the effects of miR-30d-5p on mitochondrial biogenesis and dynamics in C2C12 myotube cells. The data showed that miR-30d-5p mimics significantly suppressed the protein expression of NRF1 and mtDNA copy number in C2C12 myotubes (Figure 8A,B). Additionally, the protein expression of DRP1 associated with mitochondrial dynamics, was significantly reduced by miR-30d-5p mimics (Figure 8C,D). Conversely, transfection with miR-30d-5p inhibitors dramatically enhanced the mRNA expression of NRF1 and mtDNA copy number (Figure 8G,H), as well as markedly upregulates the protein expression of DRP1 (Figure 8I,J). However, miR-30d-5p mimics and miR-30d-5p inhibitors have no effect on the expression of MFN2 and FIS1(Figure 7C,E,F,I–L).These findings indicate that miR-30d-5p negatively affects mitochondrial biogenesis and dynamics in C2C12 myotubes.

### 2.9. MiR-30d-5p Targets SIRT1 and Regulates the Expression of PGC-1α

**Figure 9:**
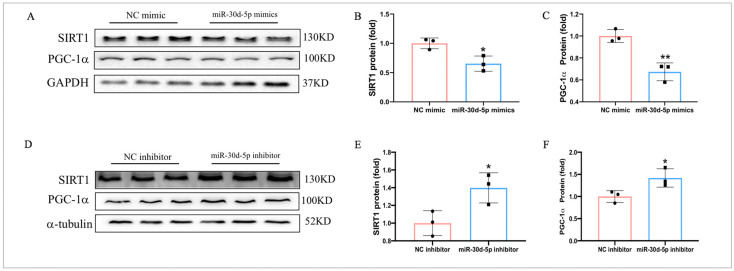
MiR-30d-5p targets SIRT1 and regulates the expression of PGC-1α. (**A**) Western blot results revealed protein levels of SIRT1and PGC-1α after transfection with miR-30d-5p mimics; (**B**) Western blot results revealed protein levels of SIRT1and PGC-1α after transfection with miR-30d-5p inhibitor; (**C**,**D**) quantification of SIRT1and PGC-1α expression levels presented in (**A**); (**E**,**F**) quantification of SIRT1and PGC-1α expression levels presented in **(B**). All data are presented as mean ± SD. *n* = 3 per group; * *p* < 0.05, ** *p* < 0.01.

To further investigate the mechanisms by which miR-30d-5p regulates glucose metabolism and mitochondrial function in C2C12 myotube cells, we examined whether miR-30d-5p targets SIRT1. The data showed that transfection with miR-30d-5p mimics significantly reduced the protein levels of SIRT1 and PGC-1α in C2C12 myotube cells (Figure 9A–C). In contrast, transfection with miR-30d-5p inhibitors significantly increased the protein levels of SIRT1 and PGC-1α (Figure 9D–F). Moreover, the previous study demonstrated that miR-30d-5p directly binds to the 3′ UTR of SIRT1. These results confirm that miR-30d-5p targets SIRT1, regulating the expression of PGC-1α and impacting glucose metabolism and mitochondrial function in C2C12 myotube cells.

## 3. Discussion

Diabetes is a serious chronic metabolic disease caused by hyperglycemia and a relative or absolute deficiency in insulin secretion. T2DM accounts for more than 90% of diabetes cases and is characterized by insulin resistance. Exercise is widely recognized as an effective approach to improve T2DM. Traditionally, aerobic exercise has been considered the gold standard for managing T2DM. However, growing evidence indicates that RE can also effectively improve glucose metabolism and insulin resistance in obese rats [14,15] and mice [16]. Human studies have also demonstrated improvements in blood glucose and glucose tolerance in T2DM patients [17], prediabetes [18], and obese [19] and overweight subjects [20] after RE intervention. Collectively, these findings underscore the potential of RE as a promising non-pharmacological approach to address obesity-related health issues, including T2DM.

Skeletal muscle plays a central role in the beneficial effects of RE on obesity-related metabolic health. As the largest insulin-sensitive tissue in the body, skeletal muscle is a primary site for glucose uptake and energy metabolism during exercise. Beyond its metabolic functions, skeletal muscle also acts as an endocrine organ by releasing myokines in response to contraction [21]. These exercise-induced myokines, including factors like irisin, IL-6, and myostatin, serve as signaling molecules that can modulate the metabolic activity of distant organs such as the liver and adipose tissue, thereby enhancing whole-body metabolic homeostasis [22,23,24]. Through these dual roles—as both an endocrine organ and a direct target of exercise—skeletal muscle contributes significantly to the systemic improvements seen with RE interventions.

In the present study, we demonstrated that eight weeks of RE effectively reduced fasting blood glucose, fat mass, serum insulin, and improved glucose and insulin tolerance in T2DM mice. Importantly, we identified the miR-30d-5p/SIRT1/PGC-1α pathway as a key mechanism through which RE improves glucose homeostasis and mitochondrial function. This suggests that RE not only enhances glucose and lipid metabolism in skeletal muscle but also potentially modulates gene expression pathways that could offer new therapeutic targets, such as miR-30d-5p, for T2DM management.

Improvement in glycemic control is related to changes in serum insulin levels, but glucose uptake is also directly related to the ability of skeletal muscle to dispose of glucose in response to insulin. The IRS-1/PI3K/Akt pathway is one of the most important signaling pathways regulating insulin resistance and insulin-mediated glucose metabolism in skeletal muscle. Previous studies have shown that the insulin signaling pathway is impaired in the skeletal muscle of db/db mice [25]. Our study found that the protein expression of IRS-1, p-PI3K/t-PI3K, and p-Akt/t-Akt was decreased in the skeletal muscle of T2DM mice compared to CON mice. Moreover, RE significantly increased the protein expression of IRS-1, p-PI3K/t-PI3K, and p-Akt/t-Akt in the skeletal muscle of T2DM-RE mice, suggesting that RE treatment improves skeletal muscle IR by activating the insulin signaling pathway, consistent with previous reports [26]. GLUT4 is the most abundant glucose transporter expressed in skeletal muscle and is mediated by the insulin signaling pathway or exercise/muscle contraction [27]. In T2DM or obese individuals, the ability of insulin to stimulate GLUT4 transport is impaired [28]. In the present study, we also found that the expression of GLUT4 was decreased in the skeletal muscle of T2DM-SED mice. Previous studies have indicated that intense progressive resistance training increased GLUT4 protein levels in the skeletal muscle of men with T2DM [5,29]. Our study’s results are consistent with these findings, indicating that RE promotes glucose homeostasis by increasing GLUT4 expression.

Furthermore, numerous studies have shown that ob/ob mice, db/db mice, or high-fat diet-fed mice have excessive lipid accumulation in skeletal muscle, which impairs insulin sensitivity. Yu et al. reported that exercise reduced lipid deposition in the skeletal muscle of high-fat diet-fed rats [30]. Our previous research also showed that high-intensity interval training (HIIT) for eight weeks effectively reduced lipid deposition in the skeletal muscle of T2DM mice [31]. Previous studies have indicated that fatty acid oxidation and the activity of enzymes involved in mitochondrial fatty acid transport and oxidation, such as CPT-1α, PPARα, and CD36, are lower in skeletal muscle during obesity and insulin resistance [32,33,34]. The results of this study indicated that the expression of PPARα decreased in the skeletal muscle of T2DM mice and increased after RE, suggesting that RE reduces skeletal muscle lipid deposition by promoting lipid oxidation. Long-chain fatty acids are the main energy source for skeletal muscles during exercise. Knockout of CD36 significantly reduces long-chain fatty acid uptake during muscle contraction/exercise [28]. Our data showed that RE significantly increased the expression of CD36 and CPT-1α, indicating that the increased CD36 may be involved in transporting fatty acids to skeletal muscle for energy during RE, and may also work with CPT-1α to transport fatty acids to mitochondria for β-oxidation. Additionally, ACCα, HMGCR, and Srebf1 are involved in lipid synthesis. This study indicated that the expression of genes related to fatty acid synthesis (e.g., HMGCR, ACCα, and Srebf1) increased in T2DM mice but reversed after RE. Abnormal lipid metabolism contributes to insulin resistance. Therefore, these results suggest that RE improves glucose homeostasis by reducing fatty acid synthesis and increasing fatty acid oxidation and transport in the skeletal muscle of T2DM mice.

Mitochondria are essential for the aerobic generation of ATP and play crucial roles in glucose, lipid, and energy metabolism. Mitochondrial-related genes regulating both mitochondrial biogenesis and dynamics may play key roles in developing insulin resistance during obesity and T2DM [35,36]. Morino et al. reported that the mitochondrial content in T2DM patients was significantly reduced [37]. Our previous research also found that the number of mitochondria in the gastrocnemius muscle of T2DM mice was significantly reduced [38]. Mitochondrial quantity is related to mitochondrial biogenesis. NRF-1 and mtDNA copy number are involved in regulating mitochondrial biogenesis. Our data indicated that mtDNA copy number and NRF-1 expression significantly decreased in the skeletal muscle of T2DM mice. Exercise affects mitochondrial quality control in skeletal muscle, mainly through transcriptional regulation, including PGC-1α, mtDNA, NRF-1, and Tfam [39]. We also found that RE significantly increased mitochondrial DNA copy number and NRF-1 expression. The decrease in mitochondrial biogenesis induces abnormal glucose and lipid metabolism in skeletal muscle. Therefore, our results suggest that RE may improve lipid and glucose metabolism in T2DM mice by regulating mitochondrial biogenesis.

Mitochondria are highly plastic organelles, and the morphology of mitochondria in the skeletal muscle of T2DM mice is abnormal, related to mitochondrial fusion and fission. A disturbed balance between fusion and fission, based on the expression of involved proteins in skeletal muscle, has been identified in obesity and T2DM [40]. MFN2 regulates the fusion of the outer membrane, while DRP1 and FIS1 regulate the involvement of the outer membrane in mitochondrial fission. Human studies have revealed that the expression of fusion proteins is reduced in the skeletal muscle of T2DM patients [41,42]. Our data showed that the protein expression of MFN2, DRP1, and FIS1 was not changed in the T2DM-SED mice compared to CON mice. This difference may be due to the difference in subjects used in the two studies. Previous studies reported that resistance exercise increased the protein expression of MFN2, FIS1, Opa1, and MFN2 in the skeletal muscles of ovariectomized rats [43] and normal rats [44]. However, few studies have indicated the effects of RE on mitochondrial fusion and fission in the skeletal muscle of T2DM mice. Our data showed that RE significantly increased the expression of DRP1, MFN2, and FIS1. Abnormal mitochondrial dynamics are associated with glucose metabolism and insulin resistance [45]. Therefore, the current study suggests that RE is a non-pharmacological treatment that promotes mitochondrial biogenesis and dynamics in T2DM mice. Improved mitochondrial dysfunction may lead to improved glucose and lipid metabolism in T2DM mice. 

It has been widely reported that T2DM is a complex pathological process, and a number of miRNAs play important roles in obesity and T2DM. To explore the mechanism of RE improving glucose homeostasis in T2DM mice, we detected the expression of multiple miRNAs after resistance exercise/mechanical stretch using qPCR. We found that the changes in miR-30d-5p are most significant in IR C2C12 myotube cells and skeletal muscle, and are reversed by cell mechanical stretch and RE. A previous study reported that the expression level of miR-30d-5p in the plasma of diabetes patients increased by 3.1 times compared to non-diabetes patients [46] and correlated with the insulin resistance index, BMI, and glycosylated hemoglobin [47]. These results indicated that miR-30d-5p is strongly associated with T2DM progression and might play an important role in the improvement of glucose homeostasis in T2DM mice by RE.

Few studies have shown altered expression of miR-30d-5p in T2DM and other metabolic diseases. Only one study reported that miR-30d-5p expression increased in the skeletal muscle of diabetic rats [47], consistent with our results. However, the mechanism by which miR-30d-5p regulates skeletal muscle glucose and lipid metabolism is still unclear. Our results demonstrated that miR-30d-5p knockdown upregulated the expression of SIRT1 and PGC-1α in C2C12 myotube cells. SIRT1 is an important deacetylase that affects the activity of various transcription factors and transcriptional regulators, including the key metabolic regulator PGC-1α. Studies have reported that SIRT1 improved glucose and lipid metabolism in diabetes by regulating PGC-1α [48,49]. PGC-1α regulates the expression of mitochondrial genes and is a key regulator of mitochondrial biogenesis and oxidative phosphorylation. We also found that inhibiting miR-30d-5p function promoted mitochondrial biogenesis and fission in C2C12 myotube cells. Moreover, SIRT1 was a direct target of miR-30d-5p. The previous study, which used a dual luciferase assay, demonstrated that miR-30d-5p could directly bind to the 3′UTR of SIRT1 [11], regulating the expression of PGC-1α and subsequently improving mitochondrial biogenesis and dynamics. We found that RE significantly suppressed miR-30d-5p expression and promoted SIRT1 and PGC-1α expression. Therefore, our data indicate that the miR-30d-5p/SIRT1/PGC-1α axis plays a key role in the improvement of mitochondrial biogenesis and dynamics after resistance exercise.

Furthermore, studies have shown that the glucose uptake capacity of trophoblast cells increased after inhibiting miR-30d-5p function [50]. Zhou et al., also found that miR-30d-5p plays a crucial role in regulating glucose metabolism by targeting the GLUT4 signaling pathway in L6 cells [51]. Our results showed that inhibiting miR-30d-5p function significantly increased glucose uptake and GLUT4 expression. PGC-1α can enhance the expression of GLUT4 to increase glucose uptake and metabolism [52]. Research has found that the SIRT1/PGC-1α/PPARα pathway plays a critical role in the regulation of β-oxidation [53]. PPARα can bind to PPRE regions in the promoters of the β-oxidation-related factors ACADM and CPT-1α, thereby promoting their transcription and facilitating fatty acid β-oxidation [54]. We found that overexpression of miR-30d-5p induced lipid deposition and inhibited fatty acid transport and oxidation, promoting fatty acid synthesis. Therefore, the data in our study indicate that the miR-30d-5p/SIRT1/PGC-1α axis plays a key role in the improvement of glucose and lipid metabolism after resistance exercise.

This study has two primary limitations. First, we did not perform a luciferase assay to confirm miR-30d’s targeting of Sirt1, as prior studies have already established this interaction. Second, we did not utilize Sirt1 knockout models to determine whether Sirt1 is a key target of miR-30d-5p in the regulation of mitochondrial biogenesis. Further studies addressing these points would provide deeper insights into the role of the miR-30d-5p/SIRT1 pathway in mitochondrial function and metabolic regulation.

## 4. Materials and Methods

### 4.1. Establishment of T2DM Mouse Models

**Figure 10:**
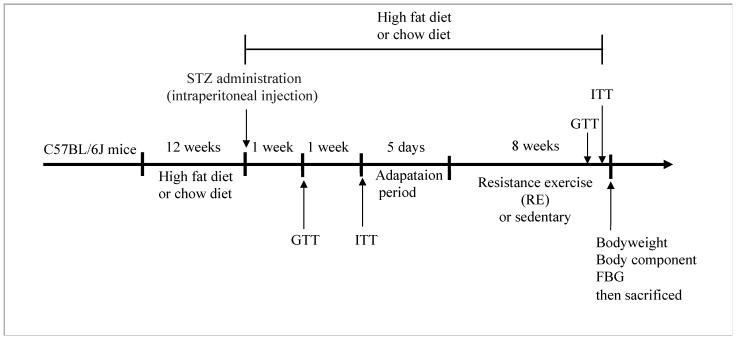
Schematic diagram of the experiment: 12 weeks of high-fat feeding was combined with intraperitoneal injection of STZ to induce a type 2 diabetes model. One or two weeks later, glucose tolerance and insulin tolerance tests were measured. Then, T2DM mice were treated with or without RE for 8 weeks.

Five-week-old male C57BL/6J mice were obtained from the Model Animal Research Center (Nanjing, China). All animals were maintained on a 12-h light/dark cycle and given free access to water. After one week of acclimatization, the mice were fed either a chow diet (3.6 kcal/g, 4.8% kcal from fat) or a high-fat diet (HFD) (Research Diet, D12492; 60% kcal from fat, 5.24 kcal/g, SYSE Ltd., Jiangsu, China) for 12 weeks. The protocol for establishing the diabetic mouse model was based on previous reports [55]. HFD-fed mice received streptozotocin (STZ, Sigma-Aldrich, St. Louis, MO, USA) at 100 mg/kg dissolved in citrate buffer (pH 4.4), while chow-fed mice received the same volume of citrate buffer. Seven days later, fasting blood glucose, glucose tolerance, and insulin tolerance were measured using a glucometer (Roche, Berlin, Germany). Mice with fasting blood glucose concentration >13.8mmol/L were considered diabetic. These T2DM mice were randomly allocated to either a resistance exercise (RE) group (T2DM-RE, *n* = 8) or a sedentary group (T2DM-SED, *n* = 8). The experimental design is shown in Figure 10. All experimental protocols were approved by the Ethics Review Committee for Animal Experimentation of the Shanghai University of Sport (Approval no. 102772023DW022).

### 4.2. Resistance Exercise Protocol

The exercise protocol for the RE mice was adapted from previous reports with slight modifications [56]. Mice in the T2DM-RE group performed training on a 1-m ladder with a 1-cm grid inclined at 85°. Before formal training, the mice underwent a 5-day adaptation protocol involving one session per day without load. Once the exercise protocol commenced, mice in the T2DM-RE group climbed from the bottom to the top of the ladder with weights attached to their tails. The weight started at 30% of the mouse’s body weight in the first week and gradually increased throughout the training period: 2nd week, 55%; 3rd and 4th weeks, 80%; 5th and 6th weeks, 90%; and 7th and 8th weeks, 100%. Training was conducted for 8 weeks, 3 days per week, five times per set, three sets per day, with 1-min rest between sets. During the 8-week RE period, mice in both the T2DM-SED and T2DM-RE groups were maintained on a high-fat diet.

### 4.3. Glucose and Insulin Tolerance Tests

The protocols for measuring fasting blood glucose, IPGTT, and ITT were based on previous reports [55].

### 4.4. Body Composition

Echo MRI (Echo Medical Systems Houston, USA) was used to assess the fat and lean body mass of mice in all groups.

### 4.5. Tissue Collection

All mice were anesthetized with isoflurane (3%, Thermo Fisher Scientific, Waltham, MA, USA) and euthanized by exsanguination. Gastrocnemius muscle and blood samples were collected. Serum was separated by centrifugation (3000 rpm, 15 min) from the blood. Gastrocnemius muscle and serum were stored at −80 °C for further analysis.

### 4.6. Determination of Serum Insulin Concentration

Fasting serum insulin levels were determined using a mouse INS ELISA kit (CEA448Mu; Cloud-Clone Corp., Houston, TX, USA) according to the manufacturer’s instructions.

### 4.7. Cell Culture and Treatment

Mouse C2C12 myoblast cells were purchased from the Cell Bank of the Chinese Academy of Sciences. They were cultured in DMEM (Thermo Fisher Scientific, Waltham, MA, USA) containing 10% fetal bovine serum (Thermo Fisher Scientific, Waltham, MA, USA) and 1% penicillin–streptomycin solution at 37 °C, 5% CO2. When C2C12 myoblast cells reached 80% confluence, the culture medium was replaced with DMEM supplemented with 2% horse serum (Thermo Fisher Scientific, Waltham, MA, USA) to induce differentiation. The medium was replaced daily, and myotubes formed after 5–6 days of differentiation for subsequent experiments.

### 4.8. Cell Transfection

MiR-30d-5p mimics, inhibitors, and negative controls were designed and synthesized by GenePharma (Shanghai, China). The sequences for the miR-30d-5p mimics were UGUAAACAUCCCCGACUGGAAG and UCCAGUCGGGGAUGUUUACAUU. The sequence for the miR-30d-5p inhibitor was CUUCCAGUCGGGGAUGUUUACA. For overexpression, C2C12 myotubes were transfected with 50 nM miR-30d-5p mimic or microRNA mimic control using Lipofectamine 2000 (Thermo Fisher Scientific, Waltham, MA, USA) for 6 h. For inhibition, C2C12 myotubes (Cell Bank of the Chinese Academy of Sciences, Shanghai, China) were transfected with 100 nM miR-30d-5p inhibitor or microRNA inhibitor control using Lipofectamine 2000 for 6 h, according to the manufacturer’s instructions. The cells and culture medium were then collected for further studies.

### 4.9. Periodic Acid–Schiff Staining

PAS staining was used to visualize glycogen. Myotube cells were washed three times after removing the culture medium, fixed with PAS Fixative (Beyotime Biotechnology, Shanghai, China) for 15 min, washed with water, air-dried, stained with periodic acid solution (Beyotime Biotechnology, Shanghai, China) for 20 min, rinsed twice with distilled water, covered with Schiff Reagent (Beyotime Biotechnology, Shanghai, China) for 15 min, and rinsed with distilled water (Beyotime Biotechnology, Shanghai, China) for 2 min. Finally, the cells were stained with Mayer Hematoxylin Staining Solution (Beyotime Biotechnology, Shanghai, China) for 2 min, washed, dried, and viewed under a brightfield microscope (magnification, ×200; Labophot-2; Nikon Corporation, Tokyo, Japan).

### 4.10. Oil Red O Staining

Oil Red O (Beyotime Biotechnology, Shanghai, China), a fat-soluble dye, was used to stain fat in cells orange-red. After removing the medium and washing the cells twice with PBS (Beyotime Biotechnology, Shanghai, China), myotube cells were fixed with Oil Red O Fixative (Beyotime Biotechnology, Shanghai, China) for 20 min, washed twice with distilled water, treated with 60% isopropanol (Sinopharm Chemical Reagent Co., Ltd., Shanghai, China) for 30 s, stained with Oil Red O Staining Solution for 15 min, rinsed with 60% isopropanol for 30 s, and rinsed three times with distilled water. Finally, cells were stained with Mayer Hematoxylin Staining Solution for 2 min and rinsed three times with water.

### 4.11. Glucose Uptake Assay

After treatment, C2C12 myotubes were starved in serum-free medium (Gibco, Thermo Fisher Scientific, Waltham, MA, USA) for 6 h. Cells were then stimulated with 100 nM insulin (Sigma-Aldrich, St. Louis, MO, USA) for 30 min, and the culture medium was collected. The glucose concentration in the culture medium was detected by the glucose oxidation method. The reaction and standard solutions were prepared according to the kit instructions (GAGO20, Sigma-Aldrich, St. Louis, MO, USA). The culture medium supernatant, glucose standard, and reaction solution were added to an ELISA plate and incubated at 37 °C for 30 min. The reaction was terminated with an equal amount of 12N sulfuric acid. The oxidized substrate was detected at A540, and the glucose concentration in each sample was calculated.

### 4.12. RNA Isolation and Quantitative Real-Time PCR

Total RNA was extracted from skeletal muscle and cells using TRIzol reagent (15596026, Invitrogen, Carlsbad, CA, USA). For miRNA, cDNA was synthesized using the Mir-X miRNA First-Strand Synthesis Kit (638313, Takara Bio Inc., Shiga, Japan) following these steps: 37 °C for 60 min and 85 °C for 5 min. For mRNA, 500 mg of total RNA was used for cDNA synthesis using the Reverse Transcription Kit (RR036A, Takara Bio Inc., Shiga, Japan) following these steps: 37 °C for 15 min and 85 °C for 15 s. The SYBR Green PCR Master Mix Kit (638314, Takara Bio Inc., Shiga, Japan) was used for relative quantification of miRNA and mRNA. Real-time PCR was performed with the QuantStudio™ 3 System (Thermo Fisher Scientific, Waltham, MA, USA) to determine the relative expression of miRNAs and mRNAs. Relative gene expression was calculated using the 2^−ΔΔCt^ method after normalization to the expression level of β-actin or U6. The primer sequences are listed in Table 1.

### 4.13. Detection of Mitochondrial DNA Copy Number by Real-Time PCR

Total DNA was extracted from the skeletal muscle and cells using a PureLink Genomic DNA Mini Kit (Invitrogen, Carlsbad, CA, USA) and quantified using a NanoDrop 2000c Spectrophotometer (Thermo Scientific, Waltham, Waltham, MA, USA). The mitochondrial DNA (mtDNA) copy number was evaluated by determining the ratio of Nd1 and Cftr and was quantified by real-time PCR. For Nd1, the forward primer was 5′TCCGAGCATCTTATCCACGC′3, and the reverse primer was 5′GTATGGTGGTACTCCCGCTG ′3. For Cftr, the forward primer was 5′ATGGTCCACAATGAGCCCAG ’3, and the reverse primer was 5′GAACGAATGACTCTGCCCCT′3. The forward and reverse primers were designed and synthesized by Shanghai Sangon Biology Engineering Technology Service (Shanghai, China).

### 4.14. Protein Extraction and Western Blotting

Protein expression in muscle and cells was determined by Western blot analysis. Muscle and cells were lysed in RIPA lysis buffer, and protein concentrations were measured using a BCA Protein Assay Kit ((Thermo Fisher Scientific, Waltham, MA, USA)). Protein samples (20 μg) were loaded and separated on 4–20% SDS-polyacrylamide gels, then transferred to PVDF membranes (Bio-Rad Laboratories, Inc., Hercules, CA, USA). The membranes were blocked with 5% non-fat milk and incubated with primary antibodies against Sirtuin 1 (SIRT1), peroxisome proliferator-activated receptor-g-coactivator-1 alpha (PGC-1α), mitofusin-2 (MFN2), fission 1 (FIS1), glucose transporter 4 (GLUT4), carnitine palmitoyl transferase-1 alpha (CPT-1α), peroxisome proliferator-activated receptor alpha (PPARα), fatty acid translocase FAT/CD36, dynamic-related protein 1 (DRP1), phosphatidylinositol-3-kinase (PI3K), phosphor-phosphatidylinositol-3-kinase (p-PI3K), insulin receptor substrate 1 (IRS-1), phospho-protein kinase B (p-Akt), protein kinase B (Akt), α-tubulin, and GAPDH at 4 °C overnight. After washing with TBST, the membranes were incubated with HRP-conjugated secondary antibodies. The protein bands were visualized using enhanced chemiluminescence (ECL, Thermo Scientific, Waltham, MA, USA). The intensity of protein bands was quantified by the ImageJ software (V1.8.0, National Institutes of Health, Bethesda, MD, USA) and normalized to α-tubulin or GAPDH. The antibodies used are listed in Table 2.

### 4.15. Statistical Analysis

All data were analyzed using GraphPad Prism 9.0 (GraphPad Software, Inc., La Jolla, CA, USA). The results are presented as means ± standard deviation (SD). Statistical comparisons among the three groups were analyzed using one-way ANOVA followed by Tukey’s post hoc test. The significance of differences was calculated using Student’s *t*-test between two groups. *p* < 0.05 was considered statistically significant.

## 5. Conclusions

In conclusion, this study identifies a novel mechanism by which RE improves glucose homeostasis and mitochondrial function in T2DM mice. We demonstrate that RE effectively enhances glucose and lipid metabolism through activation of the insulin signaling pathway, improved glucose uptake, enhanced lipid oxidation, and upregulation of mitochondrial biogenesis and dynamics. Additionally, we highlight the miR-30d-5p/SIRT1/PGC-1α axis as a central pathway mediating these beneficial effects, suggesting that miR-30d-5p may serve as a potential therapeutic target for improving metabolic health in T2DM.

## Figures and Tables

**Table 1:** Primers used for quantitative real-time PCR analysis.

Gene Name	Forward Primer Sequences	Reverse Primer Sequences
*HMGCR*	GACCAACCTTCTACCTCAGCAAGC	CCAGCCATCACAGTGCCACATAC
*ACCα*	CCCAGAGATGTTTCGGCAGTCAC	GTCAGGATGTCGGAAGGCAAAGG
*Srebf1*	GCGGTTGGCACAGAGCTTCC	CCTCCTCCTCAGACTGCGATCC
*β-actin*	ATCACTATTGGCAACGAGCGGTTC	CAGCACTGTGTTGGCATAGAGGTC
*miR-30d-5p*	CGTGTAAACATCCCCGACTGGAA	
*U6*	GGAACGATACAGAGAAGATTAGC	TGGAACGCTTCACGAATTTGCG

**Table 2:** Antibodies used for Western blotting.

Name	Brand and Item Number	Dilution Ratio
SIRT1	13161-1-AP, Proteintech, USA	1:1000
PGC-1α	66369-1-Ig, Proteintech, USA	1:500
MFN2	#9482, CST, Beverly, MA, USA	1:1000
FIS1	10956-1-AP, Proteintech, USA	1:500
GLUT4	#2213, CST, Beverly, MA, USA	1:1000
CPT-1α	15184-1-AP, Proteintech, USA	1:1000
PPARα	668261-Ig, Proteintech, USA	1:1000
CD36	AF2519, R&D, MN, USA	1:1000
DRP1	#8570, CST, Beverly, MA, USA	1:1000
PI3K	#4249, CST, Beverly, MA, USA	1:1000
p-PI3K	#17366, CST, Beverly, MA, USA,	1:1000
IRS-1	#2382, CST, Beverly, MA, USA	1:1000
p-Akt	#4058, CST, Beverly, MA, USAates	1:1000
Akt	#4685, CST, Beverly, MA, USA	1:1000
α-tubulin	#4685, CST, Beverly, MA, USA	1:1000
GAPDH	#2118, CST, Beverly, MA, USA	1:1000

## Data Availability

The original contributions presented in the study are included in the article; further inquiries can be directed to the corresponding author.
